# Sinus node dysfunction and related permanent pacemaker implantation after major cardiac surgeries, systematic review, and meta-analysis

**DOI:** 10.3389/fcvm.2023.1091312

**Published:** 2023-03-10

**Authors:** Reza Hosseini Dolama, Amir Hosein Eghbal, Malihe Rezaee, Ali Vasheghani Farahani, Arash Jalali, Kaveh Hosseini

**Affiliations:** ^1^Students’ Scientific Research Center (SSRC), Tehran University of Medical Sciences, Tehran, Iran; ^2^Tehran Heart Center, Cardiovascular Diseases Research Institute, Tehran University of Medical Sciences, Tehran, Iran; ^3^Department of Pharmacology, School of Medicine, Shahid Beheshti University of Medical Sciences, Tehran, Iran; ^4^Cardiac Primary Prevention Research Center, Cardiovascular Diseases Research Institute, Tehran University of Medical Sciences, Tehran, Iran

**Keywords:** sinus node dysfunction, permanent pacemaker, cardiac surgery, cox-maze procedure, valve surgery

## Abstract

**Background:**

There is no concise evidence or clinical guidelines regarding the incidence of sinus node dysfunction (SND) and permanent pacemaker (PPM) implantation following cardiac surgeries and their management approaches.

**Objective:**

We aim to systematically review current evidence on the prevalence of SND, PPM implantation concerning it, and its risk factors in patients undergoing cardiac surgery.

**Method:**

Four electronic databases (Cochrane Library, Medline, SCOPUS, and Web of Science) were systematically searched for articles regarding SND after cardiovascular surgeries and reviewed by two independent researchers, and a third review in case of discrepancies. Using the random-effects model, a proportion meta-analysis was performed on data regarding PPM implantation. Subgroup analysis was performed for different interventions, and the possible effect of different covariates was evaluated using meta-regression.

**Results:**

From the initial 2012 unique records, 87 were included in the study, and results were extracted. Pooled data from 38,519 patients indicated that the overall prevalence of PPM implantation due to SND after cardiac surgery was 2.87% (95% CI [2.09; 3.76]). The incidence of PPM implantation in the first post-surgical month was 2.707% (95% CI [1.657; 3.952]). Among the four main intervention groups, including valve, maze, valve-maze, and combined surgeries, maze surgery was associated with the highest prevalence (4.93%; CI [3.24; 6.92]). The pooled prevalence of SND among studies was 13.71% (95% CI [8.13; 20.33]). No significant relationship was observed between PPM implantation and age, gender, cardiopulmonary bypass time, or aortic cross-clamp time.

**Conclusion:**

Based on the present report, patients undergoing the maze and maze-valve procedures are at higher risk of post-op SND, whereas lone valve surgery had the lowest prevalence of PPM implantation.

**Systematic Review Registration:**

PROSPERO (CRD42022341896).

## 1. Introduction

With the increased number of cardiac surgeries performed worldwide, the importance of post-operative complications grow. Rhythm disturbances are common after cardiac surgeries, resulting in delayed discharge, increased health care costs, morbidity, and mortality. Among post-operative arrhythmias, Atrial fibrillation (AF) is the most frequent, followed by ventricular arrhythmias, Atrioventricular blocks, and Sinus node dysfunctions (1). The risk factors, prevention, and management of AF have been comprehensively discussed in existing guidelines and consensus documents (2). However, the same could not be said regarding Brady arrhythmias, particularly Sinus Node dysfunctions (SNDs).

Sinus Node Dysfunction most commonly results from the senescence of the sinoatrial node and its surrounding myocardium and is not as common as other post-surgery arrhythmias and usually has preexisting components (3). The reported incidence of SND varies due to different duration of cardiac monitoring and its methods from one study to another. Age, underlying Sick Sinus Syndrome, hypothermia, ischemia, and inflammation are some of the various patient and procedure-related risk factors (4, 5). Furthermore, some procedures are associated with a higher incidence of SND. Permanent pacing due to sinus node dysfunction or AV conduction disruptions is required for 0.8% to 3.4% of patients following coronary artery bypass grafting (CABG) (6). This rate can reach 20% to 24% in calcific aortic stenosis and tricuspid valve replacement (7). Such observations may result from direct surgical injury, local inflammation, or injuries caused during right atrial cannulation.

Moreover, controversies exist regarding the management of SNDs in post-operative patients. Considering the transient nature of most SNDs following cardiac surgeries, more conservative approaches such as watchful waiting, temporary epicardial pacing using wires placed during surgery, catecholamine infusion, and ionotropic medication such as theophylline and aminophylline have been proposed (3, 5, 8). However, the usual practice involves PPM implantation if severe SND persists for more than 5–7 days (9).

On the grounds of lack of straightforward suggestions in existing synthesized evidence, we aim to systematically review current evidence on the prevalence of sinus node dysfunction, PPM implantation concerning it, and its risk factors in patients undergoing cardiopulmonary bypass (CBP).

## 2. Materials and methods

We conducted a systematic review of evidence regarding the incidence and management of SND after CBP. Our search strategy and selection criteria were designed regarding three main questions. (1) How common are PPM implantations due to SND in the adult population following CBP? (2) What are pre-, intra-, and post-operative factors that influence its prevalence post-operatively? and (3) how commonly do SNDs happen after CBP?

This study was performed in concordance with the PRISMA guidelines, and the initial protocol was registered in PROSPERO (CRD42022341896) (10).

### 2.1. Search strategy

Four electronic databases (Cochran library, Medline *via* PubMed, SCOPUS, and Web of Science) were searched for relevant citations using terms and keywords referring to sinus node dysfunction and cardiac surgical procedures up to 6 June 2022, with no starting date restrictions. No limits were applied to document type, language, or date. Utilized keywords are presented in Table 1, and adopted search strategies for each database are enclosed in Supplementary Table S1.

**Table 1:** Key terms used in search strategy.

Population	Intervention	Comparison	Outcome
	Cardiac Surgical Procedures [MeSH]		Sick Sinus Syndrome [Mesh]
	Cardiac AND Surg*		Sick Sinus Syndrome
	Heart AND Surg*		Sick Sinus Node Syndrome*
	Bypass*		Sinus Nod* AND Dysfunction*
			Sinus Nod* AND disease*

“*”represents truncation in the search queries.

### 2.2. Selection of studies

All retrieved citations were first screened for eligibility criteria based on their title and abstracts, and if potentially relevant, their full text was assessed. The selection process for each article was performed by two independent reviewers (R. H, A. E, or M. R), and the status of each citation (removed/entered) and the reason for rejection was entered in a spreadsheet. In cases of discrepancy between investigators, a third reviewer assessed the citation (K. H). The eligibility criteria for studies are reported in Table 2. Our primary endpoint consisted of the occurrence of SND and PPM implantation due to SND. We Defined SND as “the occurrence of symptomatic sinus bradycardia with a rate less than 50 bpm, sinus arrest or sinus pauses longer than 3 s, symptomatic sick sinus syndrome, tachy-brady syndrome,” atrial fibrillation/flutter with a rate less than 60 bpm, or a junctional rhythm when the temporary pacing was removed (11). All-cause mortality and death due to major cardiovascular events were our secondary endpoints. The essential patient characteristics for included studies consisted of age, gender, and race.

**Table 2:** Eligibility criteria for studies.

Inclusion criteria	Exclusion criteria
Original datasets reporting number of patients who underwent cardiac surgical procedures stratified based outcome endpoints (SND and PPM implantation)	Studies that do not report pre- or post-operative arrhythmias or conduction dysfunctions (including but not limited to sinus node dysfunctions)
Studies that investigate open or minimally invasive cardiac surgeries	Transcatheter interventions, Heart transplantation, and Fontan procedures
Randomized control trials, non-randomized control trials, cohort studies, case–control studies	Studies investigating Only pediatric cardiac surgeries and congenital heart disease
Studies that involve adult (>19 years old) participants	Reviews, guidelines, consensus statements case reports, letters, editorials, book chapters
Articles Published in English	

### 2.3. Data extraction

The full text of all selected studies was obtained and data regarding study characteristics, subject characteristics (age, gender, race, reported comorbidities, reported baseline characteristics), performed procedures (use of cardiopulmonary bypass, cardiopulmonary bypass time, aortic cross-clamp time), and incidence of desired outcomes (Number of early post-op sinus node dysfunction (in-hospital), late post-op sinus node dysfunction (post-discharge), post-operation PPM implantation, and all-cause mortality) was extracted into an Excel sheet. Data were extracted from tables or figures when possible, or authors were contacted If required data was not reported in the full text. The data extraction process was carried out by two reviewers (A. E and M. R) and was checked for accuracy by another reviewer (R. H). Disagreements were resolved with the consultation of a third reviewer (K. H).

### 2.4. Quality appraisal

RoB 2: a revised tool for assessing the risk of bias in randomized trials, ROBINS-I: a tool for assessing the risk of bias in non-randomized studies of interventions, the Newcastle-Ottawa Scale (NOS) for assessing the quality of non-randomized (cohort, case–control, cross-sectional) studies, and JBI critical appraisal tool for case-series were used for the quality appraisal of selected studies (12–16).

### 2.5. Data analysis

Mean ± SD and 95% CI for baseline characteristics and outcomes of all included studies have been reported with analytic weights. Due to high heterogeneity and variation in methods of SND measurement, pooled estimates were only calculated for PPM implantations. The total prevalence was estimated in each study using data from the most extended follow-up available. The pooled prevalence values and associated 95% confidence intervals were computed using random effects models through Freeman-Tukey double arcsine transformation and back transformation using Restricted maximum-likelihood estimator for between-study variance(τ^2^) (17, 18). Both Cochran’s Q test and the I2 statistic were employed to assess the heterogeneity of the studies. We also used τ^2^ statistics estimated with the restricted maximum likelihood method to evaluate the heterogeneity of studies. A τ of 0.04 and lower was interpreted as indicating low heterogeneity, whereas a value higher than 0.36 was considered highly heterogeneous. Other intermediate values were interpreted as medium heterogeneity (19).

To determine the impact of the participant’s age, gender, left ventricle ejection fraction (LVEF), body mass index (BMI), CBP time, and aortic XCL time, we conducted a sensitivity analysis by meta-regression analyses. Subgroup analyses were conducted for different intervention groups (valve, maze, valve-maze, combined surgeries, other). Additionally, we calculated prediction ranges to give a range of anticipated prevalence for PPM implantation among people having cardiac operations. Although there is no agreed-upon definition of what constitutes a positive result in a proportional meta-analysis and the assumption that positive results are more frequently published is not necessarily valid for proportional studies, we evaluated publication bias using funnel plots and The Begg and Mazumdar test (20, 21). All statistical analyses were performed using R (version 4.2.1), packages meta version 5.5.0, metafor 3.4.0, and STATA software (version 16.0, STATA Corp., College Station, Texas).

Template data collection forms, data extracted from included studies, data used for all analyses, analytic code and any other materials used in the review is available for readers upon request from the corresponding author.

## 3. Results

### 3.1. Search results

**Figure 1:**
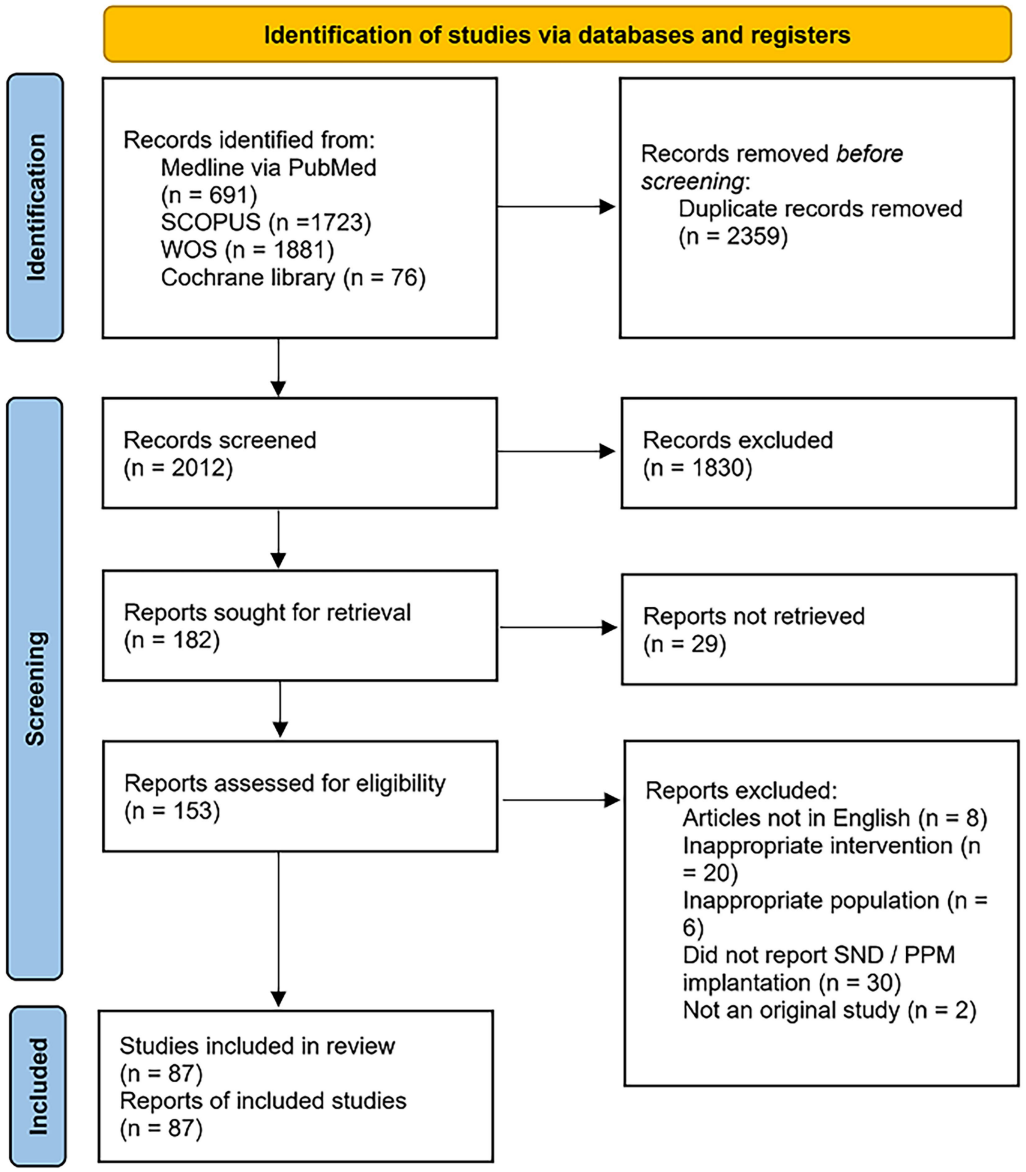
PRISMA flow diagram for the selection of included studies.

Our initial search yielded 2012 individual results, 1830 of which were excluded through the screening phase. From the remaining 182 studies, the full text of only 153 articles was available. Two independent reviewers thoroughly examined these articles and checked for inclusion and exclusion criteria, excluding 66 articles. The Preferred Reporting Items for Systematic Reviews and Meta-Analysis (PRISMA) flow diagram is presented in Figure 1.

### 3.2. Study characteristics

**Figure 2:**
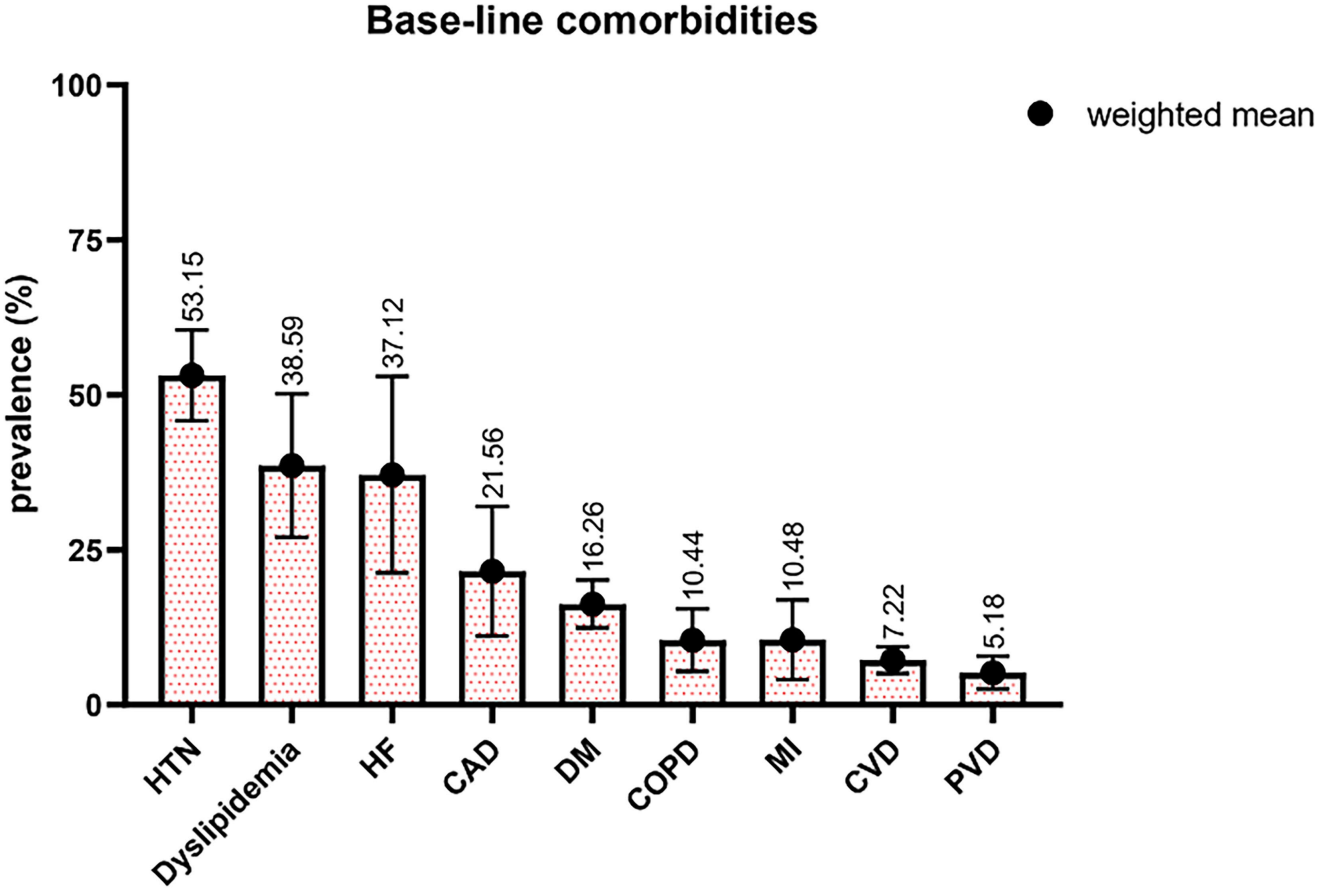
Percentages of patients with different comorbidities. HTN indicates hypertension; HF, heart failure; CAD, coronary artery disease; DM, diabetes mellitus; COPD, chronic obstructive pulmonary disease; MI, myocardial infarction; CVD, cerebrovascular disease; PVD, peripheral vascular disease.

The mean age of study participants ranged from 22.40 to 77 years, with a weighted average of 61.95 ± 7.67 years. Regarding the gender composition of studies, 36.92% ± 12.72% of patients were female. Hypertension (HTN) accounted for the most prevalent comorbidities, with a mean of 53.15% reported by 31 studies (Figure 2). It should be noted that the prevalence of heart failure (HF) was remarkedly high, 37.12%, whereas it has been reported by a few studies with all 3,246 patients. Peripheral vascular disease (PVD) was reported in 10 studies with 4,553 patients, with the least prevalence of 5.18%. Summary statistics for baseline patient and intervention characteristics and comorbidities are shown in Table 3, furthermore, a detailed description of all patient comorbidities is presented in Supplementary Table S2.

**Table 3:** Summary of baseline patient characteristics in included studies.

Variable	Studies	Participants (*n*)	Mean	Std. dev.	Min	Max	Lower 95% CI	Upper 95% CI
Age	86	24,751	61.95	7.67	22.40	77.00	60.30	63.59
Gender (female)	89	24,994	36.92%	12.72%	2.00%	88.24%	34.24%	39.60%
CPB time	57	19,532	120.81	29.57	69.12	324.52	112.96	128.65
XLC time	61	19,634	82.02	18.76	45.00	237.00	77.22	86.83
LVEF	23	4,425	54.68	3.43	47.36	63.00	53.20	56.16
BMI	14	5,938	27.42	1.90	23.90	32.11	26.32	28.52
DM	29	19,655	16.26%	10.13%	2.70%	63.89%	12.41%	20.11%
HTN	31	9,740	53.15%	19.89%	0.00%	100.00%	45.86%	60.45%
PVD	10	4,553	5.18%	3.71%	2.11%	57.14%	2.53%	7.84%
CVD	20	5,710	7.22%	4.58%	2.82%	29.41%	5.07%	9.36%
COPD	16	8,758	10.44%	9.48%	0.00%	57.14%	5.38%	15.49%
Dyslipidemia	10	3,742	38.59%	16.16%	3.45%	63.23%	27.03%	50.15%
CAD	15	6,146	21.56%	18.89%	0.00%	61.56%	11.10%	32.02%
MI	9	5,744	10.48%	8.38%	0.00%	34.85%	4.04%	16.92%
HF	11	3,246	37.12%	23.61%	9.93%	83.09%	21.26%	52.99%

### 3.3. Quality of included studies

The studies that were included were highly qualified overall. The RoB-2 tool used five separate bias assessments: reporting, performance, attrition, detection, and selection. The only RCT included had a low risk for reporting, attribution, and selection, but there were few concerns about performance and detection using the RoB-2 tool. According to the ROBINS-I tool, all studies had a low to moderate risk of bias, except for one non-RCT research with a serious risk of bias. Also, in the case series group judged by the JBI critical appraisal tool, all the studies achieved an acceptable reporting quality except for one that needed further information (22). High-quality observational studies were included, according to the Newcastle-Ottawa Scale (score > 6) used to evaluate non-randomized research. Detailed information about the quality of all included studies is presented in Supplementary Tables S3–S7.

### 3.4. Sinus node dysfunction

Forty-two studies reported the occurrence of SND after cardiac surgery, forming 48 intervention arms in total. Detailed regarding study design, interventions, follow-up, sample size, age, gender composition, cardiopulmonary bypass time, aortic cross-clamp time, and outcome measures are available in Supplementary Table S8. The weighted mean age of participants equaled 56.95 ± 11.18, ranging from 22.4 to 73.23; on average, 44.48% ± 11.70% were female. The pooled prevalence of SND among studies was 13.71% (95% CI 8.13–20.33).

### 3.5. Permanent pacemaker implantation due to SND

#### 3.5.1. Pooled analysis of overall studies

**Figure 3:**
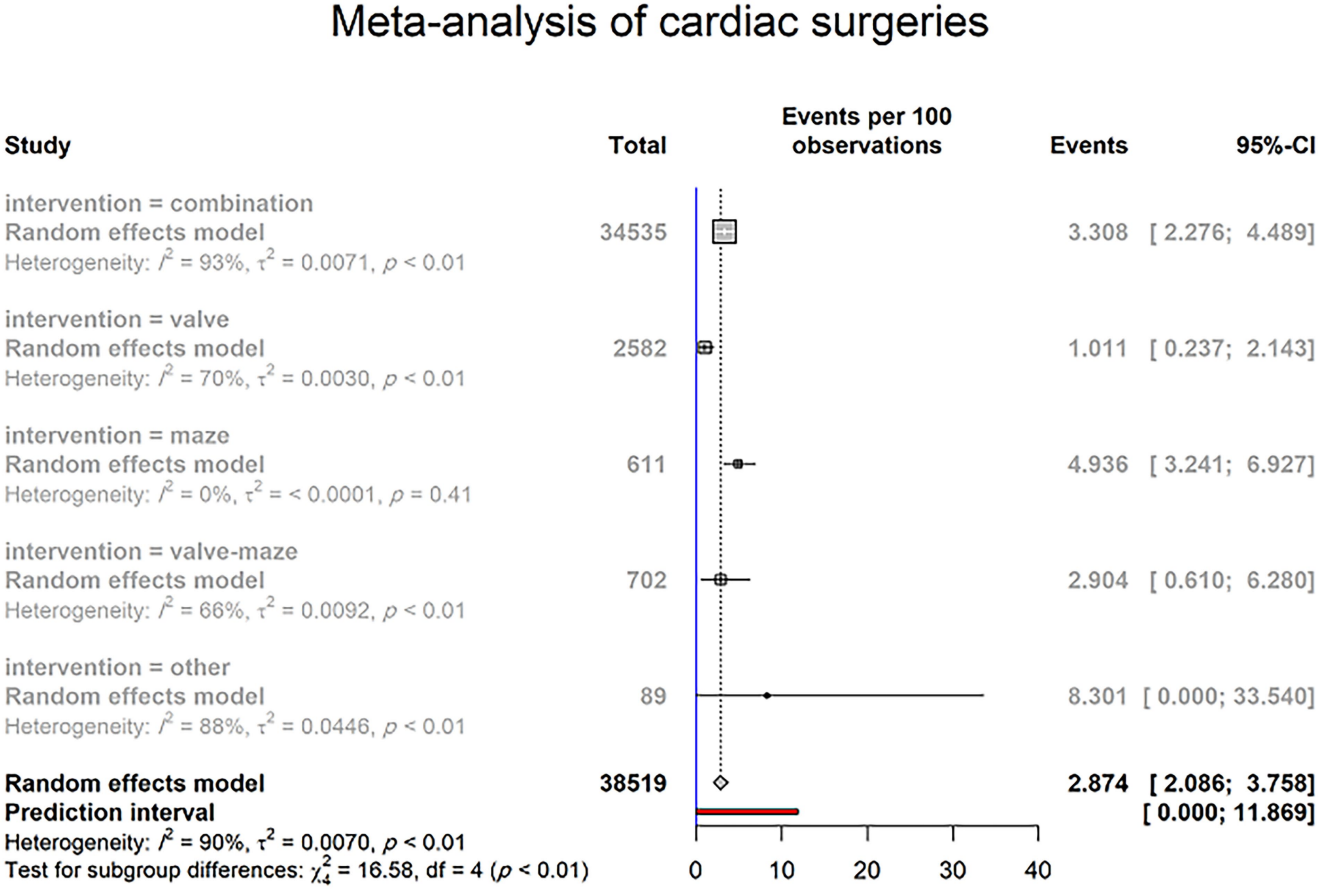
PPM implantation rate due to SND in patients undergoing cardiac surgeries (random effects model). The Events (percentages) in different intervention subgroups are represented by squares, through which the horizontal lines represent the 95% CIs. The diamond at the bottom represents the pooled intubation rate from these studies. The red bar in the bottom represents the prediction interval.

Of 87 included studies, 78 reported the PPM implantation rate resulting from SND, constituting 83 study groups with a total of 23,572 patients. The overall prevalence of PPM implantation after cardiac surgery was 2.874% (95% CI 2.086–3.758; I^2^ = 90.2%; τ^2^ = 0.0070; Cochran Q = 837.01, *p* < 0.0001; Figure 3) on random effects models. The prediction interval for the prevalence of PPM implantation ranged from 0.000% to 11.869% (Figure 3). There was a significant difference between 5 groups of cardiac surgeries in the prevalence of PPM following intervention (*p*-value = 0.0023; Figure 3).

#### 3.5.2. Subgroup and sensitivity analyses

A subgroup analysis was performed to determine the difference in PPM implantation prevalence between 5 intervention groups, including valve surgery, maze surgery, a combination of valve and maze surgery, combined cardiac surgery, and other interventions.

Thirteen studies reported PPM implantation after combined valve and maze surgery. Twenty-five of 702 patients underwent PPM implantation. Based on the random effects model, the pooled prevalence of PPM implantation after combined valve and maze surgery was 2.904% (95% CI 0.610–6.280; I^2^ = 66%; τ^2^ = 0.0092; Q = 35.35, *p* < 0.0004; Supplementary Figure S1). The prediction interval for the prevalence of PPM implantation ranged from 0.000% to 16.871%, with 95% confidence (Supplementary Figure S1).

According to the data of the five studies, 32 of 611 included patients have required to implant PPM after lone maze surgery, with the prevalence of PPM implantation following lone maze surgery was 4.936% (95% CI 3.241–6.927; I^2^ = 0%; τ^2^ < 0.0001; Q = 3.98, *p* = 0.4092; Supplementary Figure S2) on random effects model, with the 4.59% to 87.01% range of prediction interval, with 95% confidence (Supplementary Figure S2).

Regarding lone valve surgery, 14 studies with all 3,087 included patients have reported 61 cases who underwent PPM implantation. Five studies reported no cases of PPM implantation, which may refer to a limited population. Whereas a recent study reported that 21 patients were required to PPM in the larger population of 704 patients. Overall, the random effect model showed the 1.187% (95% CI 0.392–2.282; I^2^ = 71%; τ^2^ = 2.282; Q = 39.43, *p* < 0.0001; Supplementary Figure S3) prevalence of PPM implantation among patients who underwent lone valve surgery. The prediction interval for PPM implantation prevalence varied from 0.000% to 5.927%, with 95% confidence (Supplementary Figure S3).

**Figure 4:**
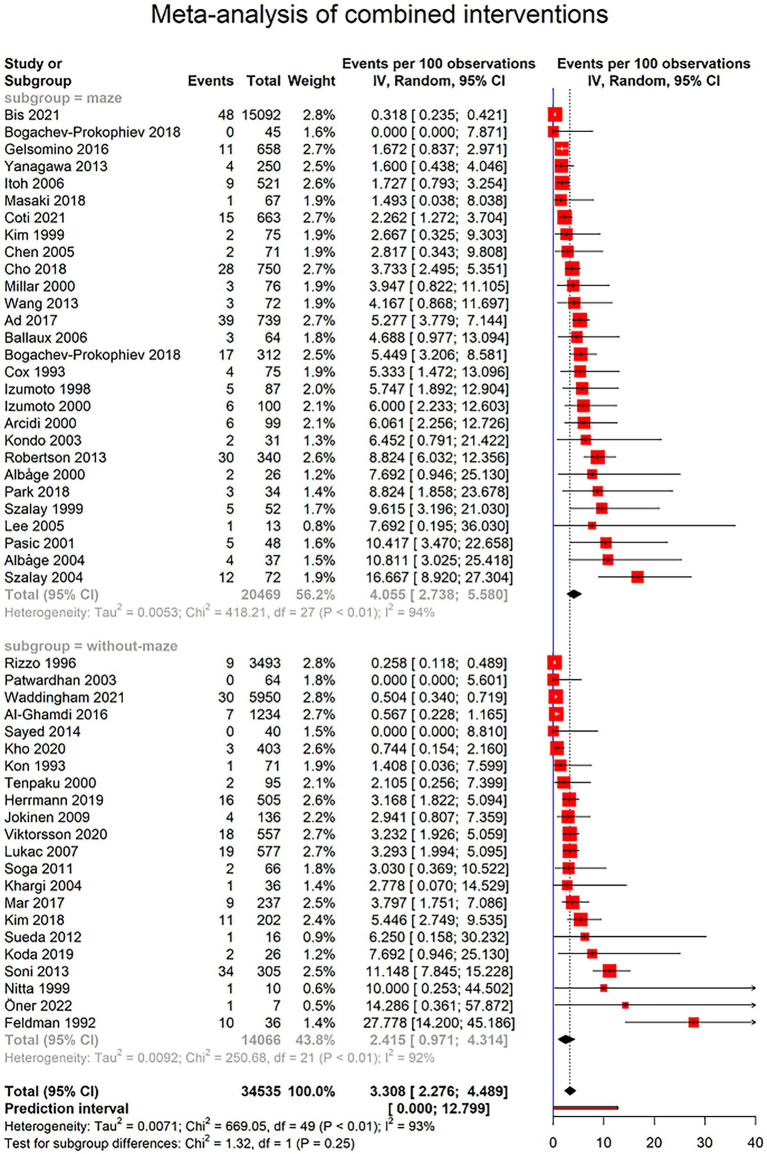
Forest plot showing pooled estimates of permanent pacemaker (PPM) implantation prevalence in patients undergoing combined cardiac surgeries using random effects model (REM). Studies have been further divided into surgeries including maze surgery and studies without maze surgery.

Figure 4 demonstrates 443 cases of PPM implantation among 34,535 patients with combined cardiac surgery from 50 studies. Three of all studies reported no cases of PPM implantation. Based on random effects model, the prevalence of PPM implantation after combined cardiac surgery was 3.308% (95% CI 2.276–4.489; I^2^ = 92.7%; τ^2^ = 0.0071; Q = 669.05, *p* < 0.0001; Figure 4), with the prediction interval of 0.000% to 12.799%, with 95% confidence (Figure 4). Moreover, studies with combined surgery divided two groups based on maze surgy, and further analysis was performed to determine the prevalence of PPM implantation in these two groups. Twenty-eight studies with total of 20,469 patients reported 270 cases who required PPM after combined cardiac surgery, including maze surgery. The prevalence of PPM implantation in this group was 4.055% (95% CI 2.738–5.580; I^2^ = 93.5%; τ^2^ = 0.005; Q = 418.21; Figure 4). However, 181 of all 14,066 patients from 22 studies reported having undergone PPM implantation after combined cardiac surgery that did not include maze surgery. The prevalence of PPM implantation in this group was 2.415% (95% CI 0.971–4.314; I^2^ = 91.6%; τ^2^ = 0.0092; Q = 250.68; Figure 4). There was no statistical difference between these two groups in the prevalence of PPM implantation (*p*-value = 0.2514; Figure 4).

Two of included studies reported PPM implantation after cardiac surgeries other than in prior groups. One study reported the PPM implantation in 6 of 30 patients following corridor surgery for atrial fibrillation (AF), and another study showed one of 59 patients who underwent hybrid ablation of AF (thoracoscopic ablation followed by catheter ablation). Accordingly, the prevalence of PPM implantation in this group was estimated 8.301% (95% CI 0.000–33.540; I^2^ = 88%; τ^2^ = 0.0446; Q = 8.2; 2 studies; 89 patients; Supplementary Figure S4).

Further sensitivity analyses were performed based on the “leave-one-out” strategy for four groups of intervention, including “valve surgery,” “maze surgery,” “combination” of valve and maze surgery, and combined cardiac surgery (Supplementary Figures S5–S8, respectively). Also, a leave-one-out analysis for all cardiac surgeries was performed (Supplementary Figure S9). The prevalence of post-surgery PPM implantation showed no change after applying sensitivity analysis in all groups.

#### 3.5.3. Timing for PPM implantation

**Figure 5:**
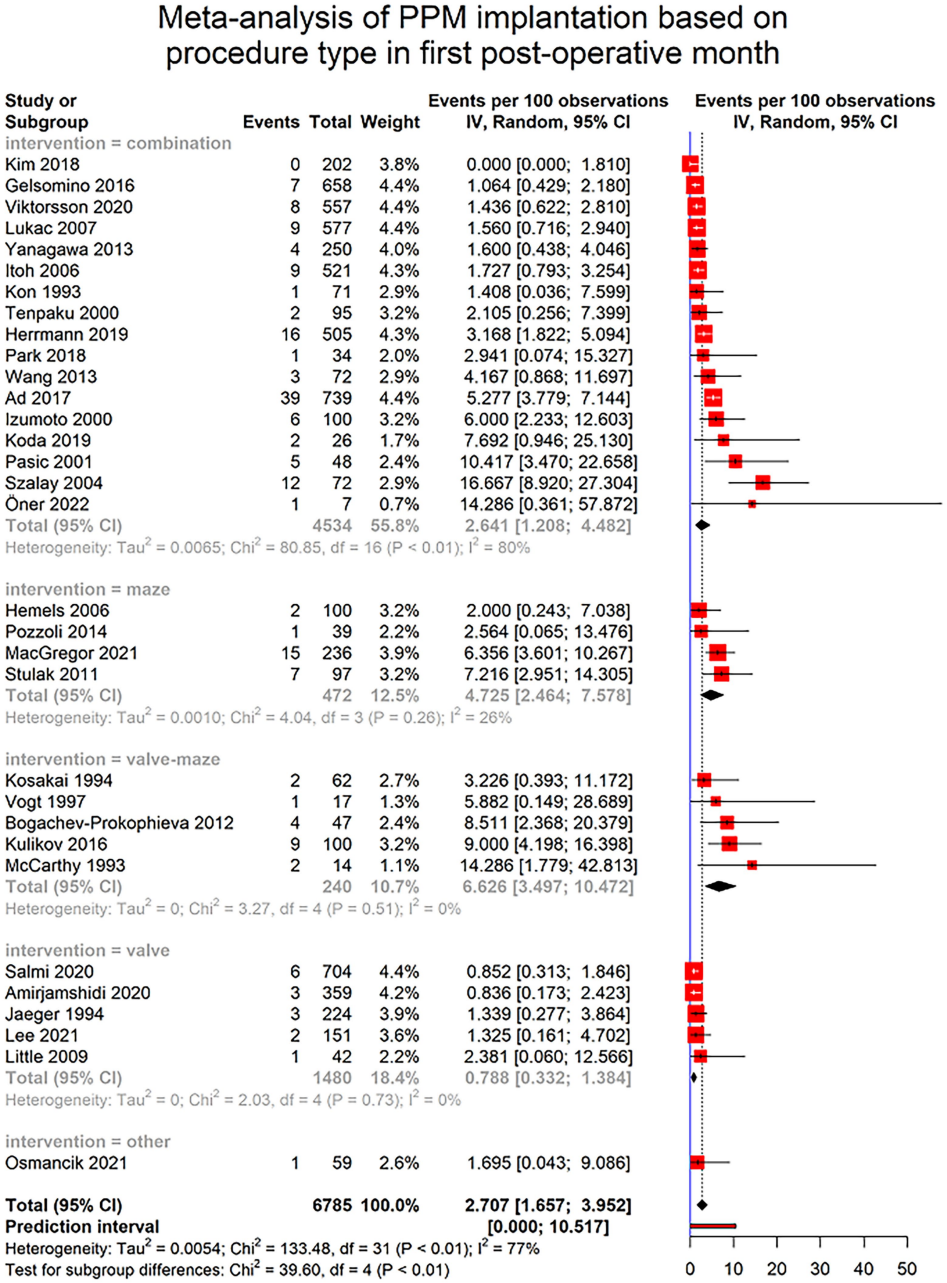
Forest plot showing pooled estimates of permanent pacemaker (PPM) implantation incidence in patients undergoing cardiac surgeries in the first month after surgery using random effects model (REM). Studies have been further divided into subgroups based on the type of the procedure.

Only 37 of included studies provided data regarding the timing of PPM implantation. Furthermore, the reported data was heterogenous among studies, with some only reporting the number of PPM implantations in the first post-surgical year without any further detail. To homogenize the data, we were able to specify the number of PPMs implanted in first post-surgical month in 32 studies. The pooled incidence of PPM implantation in first post-operative month was 2.707% (95% CI [1.65–3.95], I2 = 77%, τ^2^ = 0.0054) with a prediction interval of 0 to 10.51%. This rate significantly differed among different types of surgery (*p*-value < 0.0001). Detailed report of the PPM implantation because of SND is provided in Figure 5.

#### 3.5.4. Meta-regression

Based on our analysis, among reported variables in the included studies, five variables, including age, gender, CBP time, XCL time, EF, and BMI entered in the meta-regression analysis to determine the association between these variables and the prevalence of PPM implantation after cardiac surgeries. Results in Table 4 suggest that statistically, no significant association was detected between age, gender, CBP time, XCL time, EF, and BMI with the need for PPM implantation in patients with cardiac surgeries (all *p*-value > 0.05).

**Table 4:** Meta regression of selected variables and permanent pacemaker (PPM) implantation rate with random effects model (REM).

Variable	No. of studies	Participants (n)	Mean % (± SD)	Coefficient	*p*-value (95% confidence interval)
Age (years)	76	23,105	61.89 (±7.21)	0.000	0.93 [−0.0017–0.0019]
Gender (female ratio)	78	23,393	0.36 (±0.12)	0.069	0.23 [−0.04–0.17]
Bypass time (min)	51	18,206	122.80 (±28.97)	0.000	0.21 [−0.0001–0.0008]
Cross clamp time (min)	55	18,453	82.65 (±18.82)	0.000	0.35 [−0.0004–0.001]
%EF	22	4,106	54.73 (±3.55)	0.001	0.797 [−0.008–0.01]
BMI	13	5,014	27.44 (±2.07)	0.003	0.71 [−0.01–0.01]

#### 3.5.5. Publication bias

**Figure 6:**
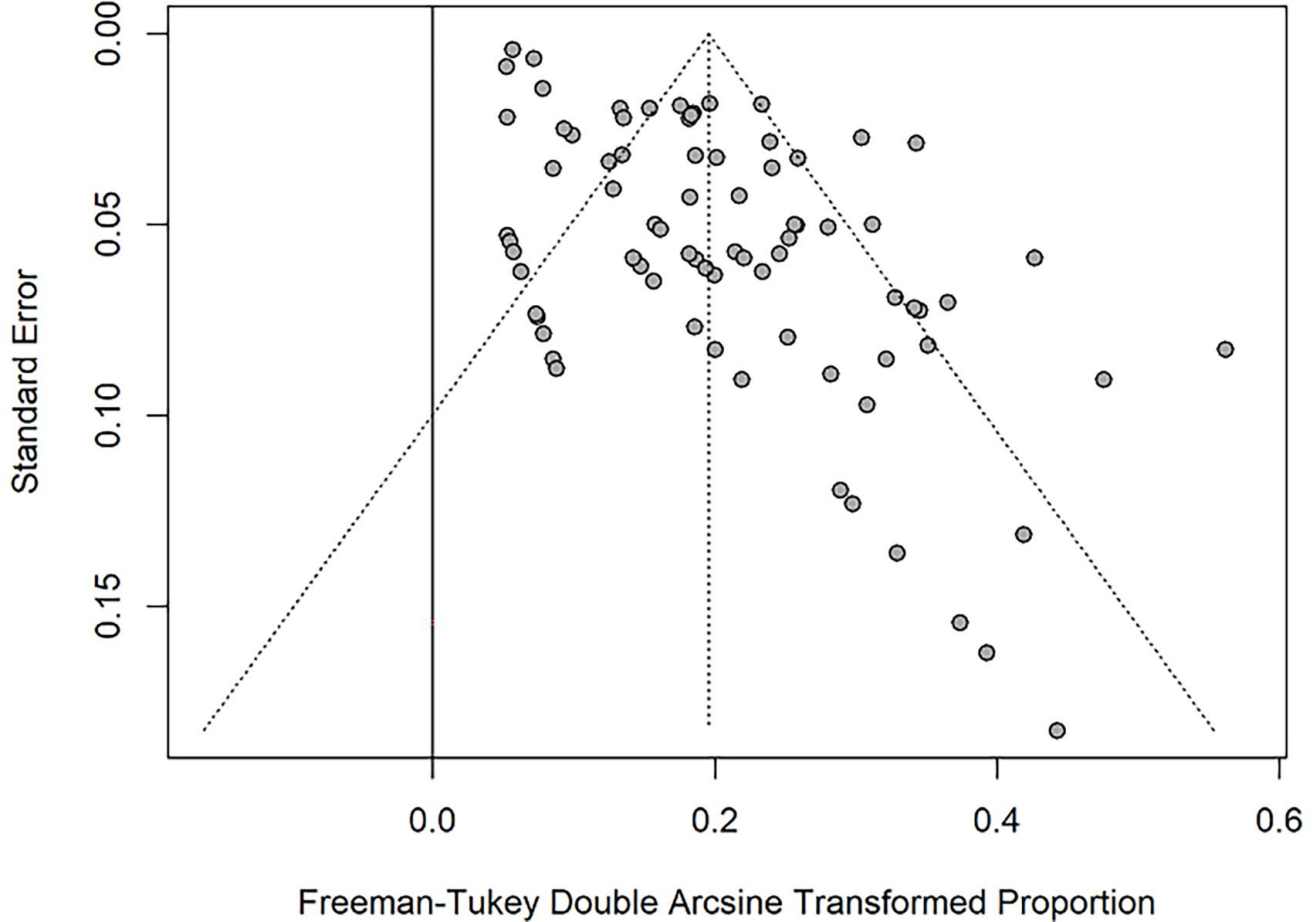
Funnel plot for the Freeman-Tukey Double Arcsine Transformation proportion of PPM across all studies.

The funnel plot for all included studies revealed no significant publication bias regarding the prevalence of PPM (Figure 6). The Begg and Mazumdar test indicate funnel plot was symmetrical (*p*-value = 0.853).

## 4. Discussion

In the current study, a systematic search, review, and meta-analysis of literature were conducted to estimate the prevalence of SND and PPM implantation due to SND following cardiac surgeries. As the prevalence and incidence of aforementioned events in adults without anatomical abnormalities have not been a subject for evidence synthesis and a lack of recommendations regarding the importance and management approaches for SND exists in guidelines and consensus statements, this study may pose added value in this domain (23–25).

SND, previously known as sick sinus syndrome (SSS) are disorders in the sinoatrial node or its surrounding tissue that affect the creation or conduction of electrical impulses. Age is the most common and significant risk factor associated with SND (26). Other conditions associated with older age, including diabetes mellitus (DM), coronary heart disease, hypertension, chronic kidney disease are also overlapping risk factors of SND. Etiology of SND can be classified as intrinsic (e.g., Cardiomyopathies, connective tissue disorders, infiltrative disease, or post-surgical changes) and extrinsic (e.g., DM, metabolic abnormalities, medication) (26, 27).

The underlying pathophysiology for SND following cardiac surgeries is yet unknown, however it is likely to include direct surgical damage with subsequent cell edema and localized myocardial ischemia (28). Existing literature suggest that the use of a right lateral atriotomy in minimally invasive mitral valve procedures or other transseptal superior approaches to the mitral valve might result in SSS with persistent symptomatic sinus bradycardia or junctional rhythms necessitating permanent pacing (6). Age has been associated with some post-operative arrhythmias such as atrial fibrillation, however, to the best of our knowledge age has not been associated with post-operative SND (6).

### 4.1. Permanent pacemaker implantation

From 87 studies that entered the review, 78 were eligible for meta-analysis, constituting a total number of 83 intervention groups. The results of this study suggest that 2.874% (95% CI 2.09–3.76) of patients undergo PPM implantation due to SND after cardiac surgeries, with significant variation between different interventions (*p*-value = 0.0023). The results of the meta-regression suggested no significant relationship between PPM implantation and CPB time (*p* = 0.21) or XCL time (*p* = 0.35) which indicates that this variation has not been a result of differences in CPB time or XCL time. Moreover, no significant correlation was found between PPM implantation patients’ age, gender, LVEF, and BMI. Since this meta-regression was conducted on population levels because these factors had not been separately reported for participants with and without PPM, a more robust analysis was not feasible.

### 4.2. Timing for PPM implantation

The incidence of PPM implantation due to SND in the first month after surgery 2.707% (95% CI [1.65; 3.95]), which does not significantly differ from the overall prevalence of PPM implantation (2.874% [95% CI 2.09–3.76]). This may indicate the higher risk of PPM implantation in first post-surgical month. However, this interpretation may be compromised by the fact that many of included studies had only reported PPMs implanted in first month and did not further follow-up patients. This was the case in 32 of 78 studies. Comparing different surgery types, patients who underwent lone valve surgeries had the lowest incidence (0.789% [0.332; 1.384]), whereas combined valve and maze surgeries had the highest incidence of PPM implantation (6.626% [3.497; 10.472]; Figure 5).

### 4.3. Maze surgery

Among the four main intervention groups, lone maze surgery was associated with the highest prevalence of post-operation PPM implantation (4.93% CI [3.24; 6.92], Figure 3) (29–33). This might be due to the nature of the maze surgery, in which lesions are imposed on atria that lead to conduction disorders in tissues surrounding the sinus node, hence sinus node dysfunction (34). A higher incidence of PPM implantation in combined surgeries that include maze procedures in comparison to those without maze also favors this interpretation (5.174% vs. 2.415%, *p*-value = 0.0594, Figure 4). This observed insignificant *p*-value may be due the diluted effect of the maze procedure in studies that report combined cardiovascular surgeries, because in these studies all participants are not subjected to the maze procedure.

Furthermore, the pooled population of “lone maze” studies are significantly more male dominant in comparison to “valve,” “valve-maze,” and “combination” groups (84.08% CI [69.39; 94.72] vs. 48.40% CI [41.92; 54.91], 48.27% CI [43.26; 53.30], and 54.95% CI [50.66; 59.21], respectively). Findings of other meta-analyses regarding pacemaker implantation after transcatheter aortic valve replacement suggest that men are more likely to receive a PPM, and such a trend may also exist in maze procedures or cardiac surgeries (35, 36). However, to the best of our knowledge, such analysis has not been performed on PPM implantation after the maze procedure. As for the incidence of sinus node dysfunction, this outcome was only reported in 2 studies with values of 4.32% and 14.63% (29, 37).

### 4.4. Valve surgeries

The pooled prevalence of PPM implantation in 2,582 patients undergoing lone valve surgery from 13 studies equaled 1.01% CI [0.23; 2.14], making it the lowest prevalence among all intervention groups. The primary intervention in these studies was aortic valve replacement in 3, mitral valve surgeries in 6, and multiple valve procedures in 4 studies. The largest study in this group was Salmi 2020, with 704 participants, which focused on isolated aortic valve replacement with bioprostheses (38). Cleveland et al. reported the highest PPM implantation prevalence among these studies, with 6 of 50 patients receiving PPM (39). Seven studies have reported the prevalence of SND with a weighted average of 7.69 ± 10.42, ranging from 0 in studies by Guiraudon et al. and Tuinenburg et al. to 31.46% in a study by Kumar et al. (40–42). The largest study in this group used Rapid-deployment Intuity® and conventional bioprostheses for aortic valve replacement in 924 patients, leading to 0.86% incidence of SND (43).

### 4.5. Operations with valve and maze procedures

Thirteen studies reported the incidence of PPM implantation following operations with concomitant valve and maze procedures, establishing an aggregate prevalence of 2.90% CI [0.61; 6.28], with prevalence ranging between 0% and 14.28% (44–46). Among included studies, 12% of the weight was appointed to a study with a prevalence of 0.49% for PPM implantation (47). Based on reported data from 8 studies and 360 participants, a weighted average of the incidence of SND in the “valve-maze” group equaled 10.55% ± 19.87% (41, 44–46, 48–51).

### 4.6. Combined cardiac surgeries

Combined cardiac surgeries were the most common intervention among all entered studies, with 50 intervention groups reporting PPM implantation in 34535patients and 29 studies reporting SND. The pooled estimate for the prevalence of post-operative PPM implantation was 3.308% CI [2.28; 4.489], with a range of 0% to 27.78% (44, 45, 52). One study by Feldman et al. reported a prevalence of 27.78%, which is an outlier compared to studies with a similar population. This was probably because it only reported the characteristics of patients that had received a PPM post-operatively since it aimed to evaluate pacemaker dependency. This magnifies the prevalence in comparison to the regular target population (52). However, the sensitivity analysis suggests that omitting this study would not significantly influence the pooled estimate of the analysis (Supplementary Figure S8).

Four of these studies had populations of more than 1,000 patients. Waddingham et al. screened 5,950 patients for Cardiac implantable electronic device (CIED) implantation after cardiac surgery, during the same admission, from 2015 to 2018 and reported 250 implants, 30 of which were due to sinus node dysfunction (PPM prevalence 0.54% CI [0.34–0.72]) (53). Rizzo et al. followed 3,493 patients for 33 months on average and identified that 9 of 45 patients received post-operative PPM due to Sick sinus syndrome (54). From 1,234 patients who underwent concomitant CABG and valve replacement surgery, Al-Ghamdi and colleagues identified 20 patients who received PPM (55). The indication was sick sinus syndrome with symptomatic bradycardia in three (15%) and atrial fibrillation (AF) with a slow ventricular rate in four (20%), which, based on our definition, are classified as sinus node dysfunction (55). Finally, Bis et al. reported 185 cases of PPM implantation following cardiac surgeries in 15,902 patients, over 11 years. However, only 48 of these cases fitted our definition of SND. Unfortunately, no report of timing for PPM implantation was provided in their paper (56).

The subgroup analysis of combined cardiac surgeries based on their inclusion of the maze procedure demonstrates a lower prevalence of PPM implantation in the patients spared from the maze procedure; however, this was not statistically significant (combined with maze: 4.05% CI [2.74; 5.58]; combined without maze: 2.41% CI [0.97; 4.31]; *p*-value = 0.2514). As mentioned before, in combined surgeries, all the participants in a study may not have undergone maze surgery along with other procedures; the observed effect is diluted.

The weighted average of SND’s prevalence in the combined surgeries was 9.34% ± 14.10% with a range of 0 to 80. From these 29 intervention groups, four groups were outliers on the higher end of the range. The detailed results of these studies are further explained. From 15 patients followed by Pasic et al., 12 manifested sinus node dysfunction in the form of severe sinus bradycardia, sinus pauses or sinus arrest, sinoatrial exit block, atrial tachyarrhythmias, alternating periods of atrial bradyarrhythmias and tachyarrhythmias during the first 3 months after the surgery (57).

In another study from Pasic et al., 36 patients demonstrated sinus node dysfunction post-operatively, receiving temporary pacing using epicardial wires. Furthermore, five of these patients received a PPM afterward (58). Szalay et al. identified 38 out of 52 patients with SND in the form of junctional rhythm, 8 of which were classified as PPM dependent during the follow-up (59). In another study, 141 patients with valvular heart disease and coronary heart disease complicated with atrial fibrillation under combined intervention: using penetrating technique radiofrequency exposure to treat their atrial fibrillation (60). Forty-one percent of these patients showed signs of SND early after the operation (60).

Comparing combined interventions with and without the maze procedure, the weighted average of SND prevalence in the former group was higher than in the latter (12.32 ± 17.86 and 6.73 ± 9.46, respectively); however, this was not statistically significant (*p*-value = 0.303).

### 4.7. Limitation and direction for future studies

The limitations of the selected research limit our investigation. To limit the domination of larger studies on overall results, we used random effect models to weight the studies. The results of the current study might have been influenced by significant clinical and statistical heterogeneity. Factors such as various methodological designs, clinical settings, and patient characteristics may have contributed to this outcome. However, such factors are inseparable components of the nature of meta-analysis of proportions (61). In the current study, heterogeneity may lie in the methodological diversity of the studies and great variety in performed interventions, which is expected in prevalence data. Nevertheless, the results of the leave-one-out analysis suggest that no single study influenced the overall results of the meta-analysis. The bias from observational studies and the lack of a control group might have been another source of bias in our review. Moreover, as identification of SND and PPM implantation had not been the primary outcome of many studies, and it had only been reported as a secondary or tertiary outcome, we faced many missing data that limited the robustness of our interpretation. No evidence of publication bias was observed. As a result, the findings should be carefully considered and need additional research.

### 4.8. Conclusion

The guideline is silent about the incidence rate of SND and related PPM implementation after major cardiovascular surgeries. Based on the present report, patients undergoing the maze and maze-valve procedures are at higher risk of post-op SND. We did not find a statistically significant relationship between age, gender, CBP time, and XCL time.

## Data availability statement

The original contributions presented in the study are included in the article/Supplementary material, further inquiries can be directed to the corresponding author.

## Author contributions

RH designed the search strategy, and participated in the screening, data extraction, analysis, and manuscript drafting. AE participated in the screening, data extraction, analysis, and manuscript drafting. MR participated in the screening, data extraction, analysis, and manuscript drafting. AF provided guidance throughout the process and revised the final draft. AJ provided guidance regarding the meta-analysis of results and revised the manuscript draft. KH participated in the development of the idea, search strategy, supervised the screening, data extraction, analysis, and revised the final manuscript. All authors contributed to the article and approved the submitted version.

## Conflict of interest

The authors declare that the research was conducted in the absence of any commercial or financial relationships that could be construed as a potential conflict of interest.

## Publisher’s note

All claims expressed in this article are solely those of the authors and do not necessarily represent those of their affiliated organizations, or those of the publisher, the editors and the reviewers. Any product that may be evaluated in this article, or claim that may be made by its manufacturer, is not guaranteed or endorsed by the publisher.

## Supplementary material

The Supplementary material for this article can be found online at: https://www.frontiersin.org/articles/10.3389/fcvm.2023.1091312/full#supplementary-material
