# Supplementary material for: Sinus node dysfunction and related permanent pacemaker implantation after major cardiac surgeries, systematic review, and meta-analysis
Source: Front Cardiovasc Med. 2023 Mar 10;10:1091312. doi: 10.3389/fcvm.2023.1091312 (PMC10037194; doi:10.3389/fcvm.2023.1091312)
Supplement: Supplementary file 1 [file Data_Sheet_1.docx]

Supplementary Material

# Supplementary Figures and Tables

## Supplementary Figures


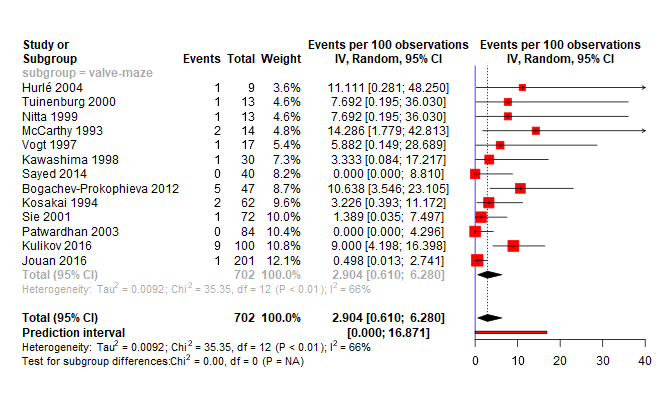


Supplementary Figure 1- Forest plot showing pooled estimates of permanent pacemaker (PPM) implantation prevalence in patients undergoing combined valve and maze surgeries using random effects model (REM).


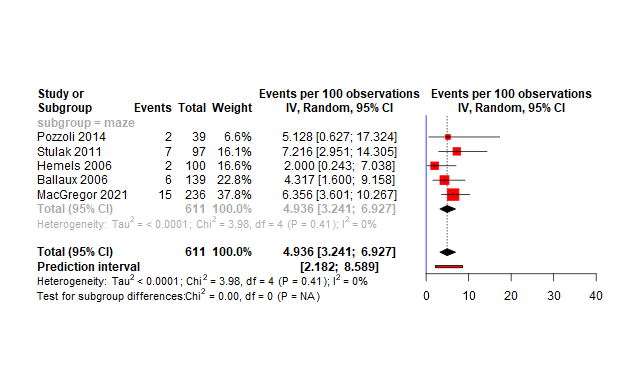


Supplementary Figure 2 - Forest plot showing pooled estimates of permanent pacemaker (PPM) implantation prevalence in patients undergoing lone maze surgery using random effects model (REM).


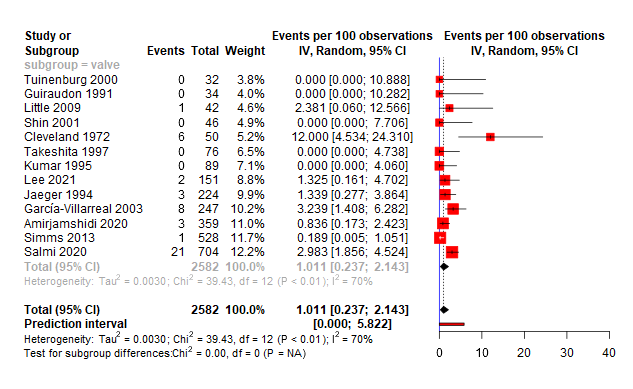


Supplementary Figure 3 - Forest plot showing pooled estimates of permanent pacemaker (PPM) implantation prevalence in patients undergoing lone valve surgery using random effects model (REM).


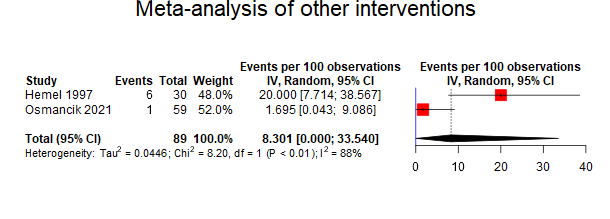


Supplementary Figure 4 - Forest plot showing pooled estimates of permanent pacemaker (PPM) implantation prevalence in patients undergoing other cardiac surgeries using random effects model (REM).


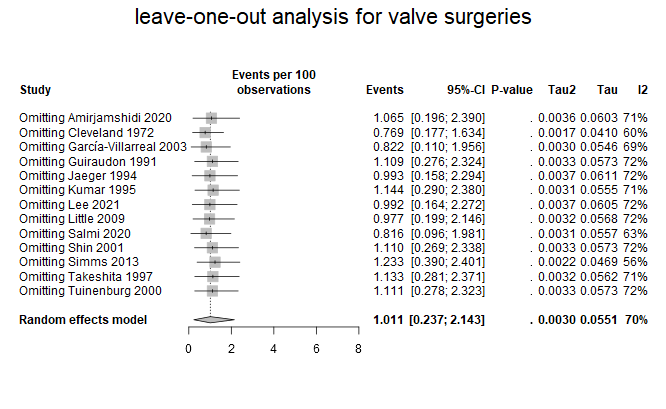


Supplementary Figure 5 - Sensitivity Analysis on the pooled estimate of PPM implantation in patients undergoing valve surgeries.


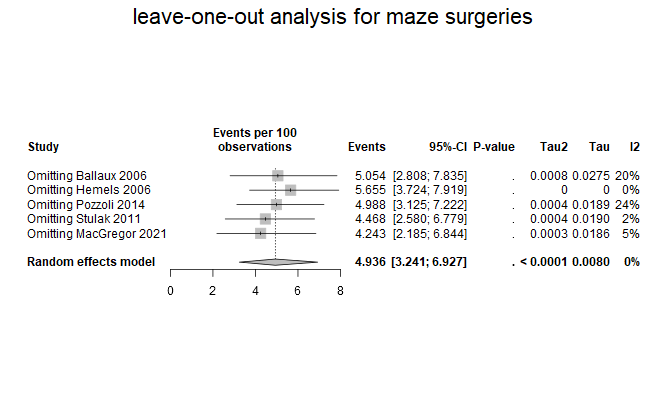


Supplementary Figure 6 - Sensitivity Analysis on the pooled estimate of PPM implantation in patients undergoing maze surgeries


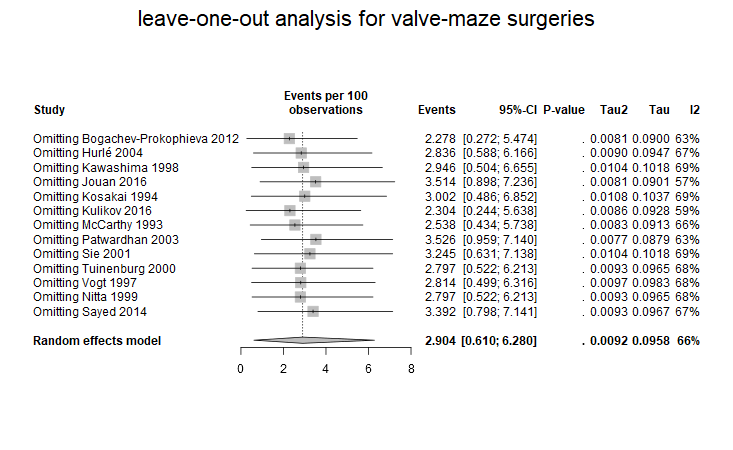


Supplementary Figure 7 - Sensitivity Analysis on the pooled estimate of PPM implantation in patients undergoing combined valve and maze surgeries.


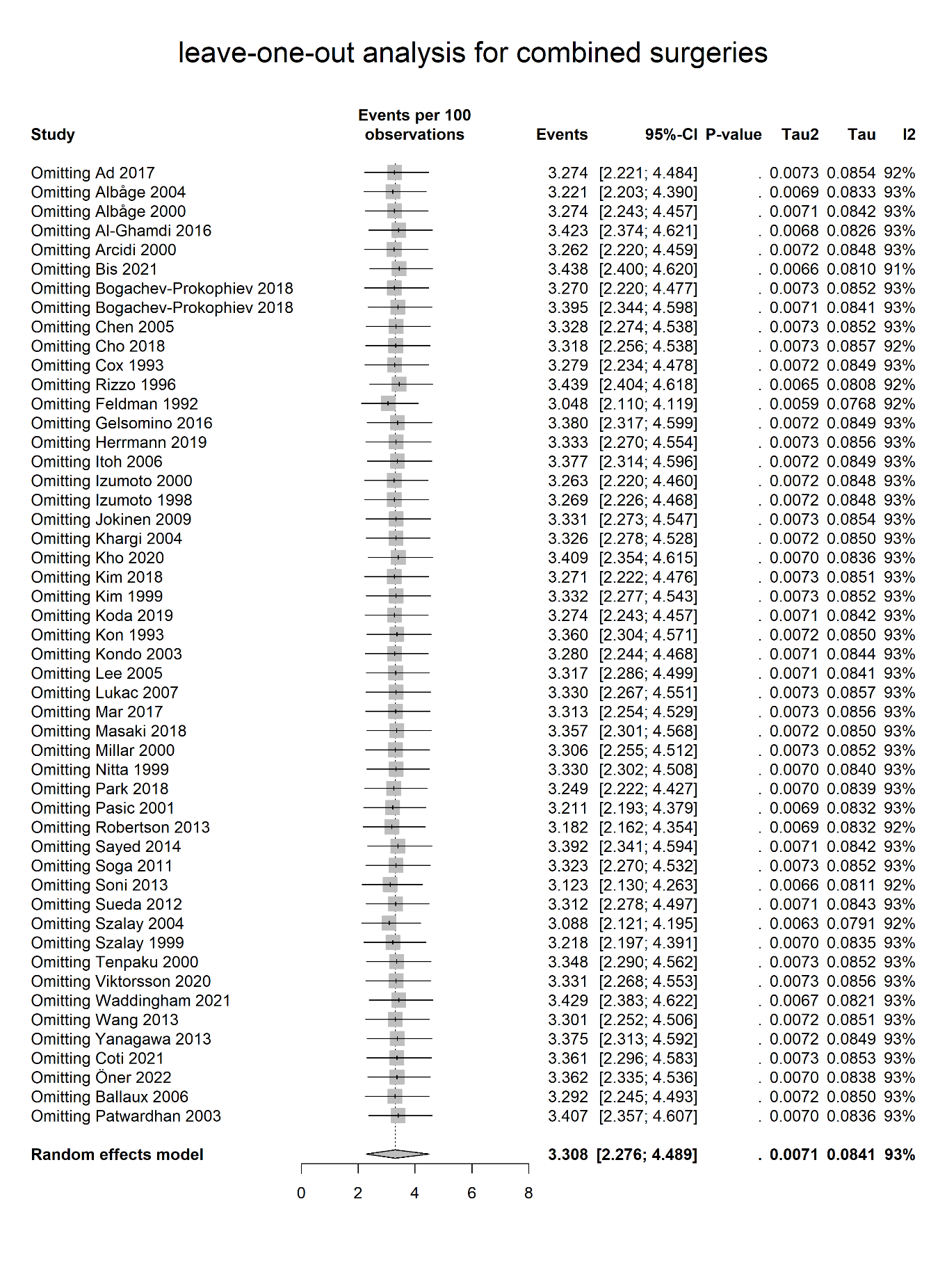


Supplementary Figure 8 - Sensitivity Analysis on the pooled estimate of PPM implantation in patients undergoing combined cardiac surgeries


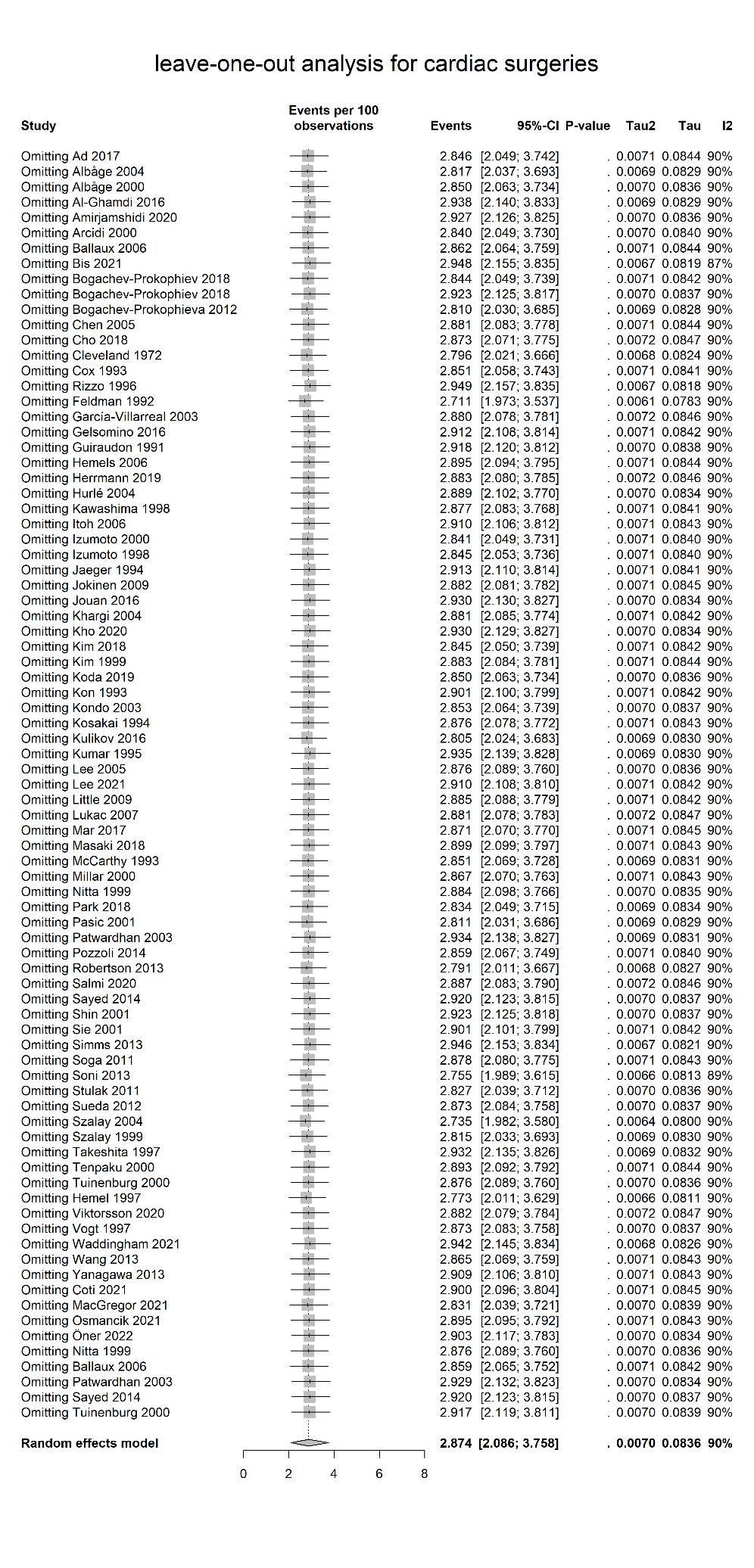


Supplementary Figure 9 - Sensitivity Analysis on the pooled estimate of PPM implantation in patients undergoing cardiac surgeries

## Supplementary Tables

Supplementary Table 1 - Search queries for different databases

| **Search query** | | **Number of results** |
| --- | --- | --- |
| **PubMed** | |  |
|  | #1 AND #2 | 691 |
| #2 | "Sick Sinus Syndrome"[MeSH Terms] OR ("Sick Sinus Syndrome"[Title/Abstract] OR "sick sinus node syndrome*"[Title/Abstract] OR ("Sinus Nod*"[Title/Abstract] AND "dysfunction*"[Title/Abstract]) OR ("Sinus Nod*"[Title/Abstract] AND "disease*"[Title/Abstract])) | 5,871 |
| #1 | "cardiac surgical procedures"[MeSH Terms] OR (("Cardiac"[Title/Abstract] AND "Surg*"[Title/Abstract]) OR ("Heart"[Title/Abstract] AND "Surg*"[Title/Abstract]) OR "bypass*"[Title/Abstract]) | 441,003 |
| **SCOPUS** | |  |
|  | #1 AND #2 | 1,723 |
| #2 | TITLE-ABS-KEY ( "Sick Sinus Syndrome"  OR  "sick sinus node syndrome*"  OR  ( "Sinus Nod*"  AND  dysfunction* )  OR  ( "Sinus Nod*"  AND  disease* ) ) | 11,447 |
| #1 | TITLE-ABS-KEY ( ( cardiac  AND surg*)  OR  (heart  AND surg*) OR Bypass*) | 611,635 |
| **Web of science (All data bases including BIOSIS, Medline, KCI, SciELO)** | |  |
|  | #1 AND #2 | 1,881 |
| #2 | TS=( "Sick Sinus Syndrome"  OR  "sick sinus node syndrome*"  OR  ( "Sinus nod*"  AND  dysfunction* )  OR  ( "Sinus nod*"  AND  disease* ) ) | 9,299 |
| #1 | TS=( ( cardiac  AND surg*)  OR  (heart  AND surg*) OR Bypass*) | 786,452 |
| **Cochrane library** | | |
|  | (#1 OR #2) AND (#3 OR #4) | 76 |
| #4 | ("Sick Sinus Syndrome"  OR  "sick sinus node syndrome*"  OR  ( "Sinus Node"  AND  dysfunction* )  OR  ( "Sinus Node"  AND  disease* )):ti,ab,kw | 579 |
| #3 | MeSH descriptor: [Sick Sinus Syndrome] explode all trees | 165 |
| #2 | ( ( cardiac AND surg*) OR (heart AND surg*) OR Bypass*):ti,ab,kw | 52,007 |
| #1 | MeSH descriptor: [Cardiac Surgical Procedures] explode all trees | 13,470 |

Supplementary Table 2 - The baseline comorbidities of the population in all the included studies (all numbers indicate percentages)

| **Study** | **DM** | **HTN** | **PVD** | **CVD** | **COPD** | **Dyslipidemia** | **CAD** | **MI** | **HF** |
| --- | --- | --- | --- | --- | --- | --- | --- | --- | --- |
| Ad 2017 | 13.4% | 61.7% | 5.5% | 6.4% | 18.7% |  |  |  |  |
| Albåge 2004 | 0.1% | 0.4% |  |  |  |  |  |  |  |
| Albåge 2000 |  |  |  |  |  |  |  |  |  |
| Al-Ghamdi 2016 | 71.6% | 78.8% | 3.5% | 5.8% |  | 45.1% | 0.0% |  |  |
| Amirjamshidi 2020 | 13.1% | 38.2% | 5.8% | 3.0% | 7.3% | 30.7% | 29.9% | 6.0% | 8.0% |
| Arcidi 2000 |  |  |  |  |  |  |  |  |  |
| Ballaux 2006 |  |  |  | 1.4% |  |  |  |  |  |
| Bogachev-Prokophiev 2018 |  | 8.5% |  |  |  |  | 5.4% |  |  |
| Bogachev-Prokophiev 2018 |  |  |  |  |  |  |  |  |  |
| Bogachev-Prokophieva 2012 | 0.3% | 1.5% |  | 0.5% |  |  |  |  |  |
| Chen 2005 |  |  |  | 0.3% |  |  |  |  |  |
| Cho 2018 | 9.3% | 26.3% |  | 4.6% |  |  | 6.1% |  |  |
| Cleveland 1972 |  |  |  |  |  |  |  |  |  |
| Cox 1995 |  |  |  |  |  |  |  |  |  |
| Cox 1993 |  |  |  |  |  |  |  |  |  |
| Rizzo 1996 | 89.3% |  |  |  | 24.1% |  |  | 37.8% |  |
| Evtushenko 2015 |  |  |  |  |  |  |  |  |  |
| Feldman 1992 | 3.1% |  |  |  |  |  |  |  |  |
| García-Villarreal 2003 |  |  |  |  |  |  |  |  |  |
| Gelsomino 2016 | 22.1% | 60.9% |  |  | 33.2% |  | 13.9% |  |  |
| Guiraudon 1991 |  |  |  |  |  |  |  |  |  |
| Hemels 2006 |  | 0.4% |  |  |  |  |  |  |  |
| Herrmann 2019 | 11.1% |  | 1.8% |  | 8.8% |  | 21.9% |  | 51.4% |
| Herry 2020 | 24.5% | 59.9% |  |  | 5.5% |  |  |  | 28.0% |
| Hurlé 2004 |  |  |  |  |  |  |  |  |  |
| Kawashima 1998 |  |  |  |  |  |  |  |  |  |
| Itoh 2006 |  |  |  |  |  |  |  |  |  |
| Izumoto 2000 |  |  |  |  |  |  |  |  |  |
| Izumoto 1998 | 0.5% | 1.8% |  |  |  | 0.4% |  |  | 8.0% |
| Izzat 2020 |  |  |  |  |  |  |  |  |  |
| Jaeger 1994 |  |  |  |  |  |  |  |  |  |
| Jessurun 2000 |  |  |  |  |  |  |  |  |  |
| Jokinen 2009 |  | 9.7% |  | 0.7% |  |  |  |  | 15.3% |
| Jouan 2016 |  |  |  |  |  |  |  |  |  |
| Khargi 2004 |  |  |  |  |  |  |  |  |  |
| Kho 2020 | 16.0% | 30.9% | 2.8% |  | 3.8% | 15.2% |  |  | 14.9% |
| Kim 2018 | 3.7% | 8.9% |  |  |  |  | 1.8% |  |  |
| Kim 1999 |  |  |  |  |  |  |  |  |  |
| Koda 2019 |  | 1.4% |  |  |  |  |  |  |  |
| Kon 1993 |  |  |  |  |  |  |  |  |  |
| Kondo 2003 |  |  |  |  |  |  |  |  |  |
| Kosakai 1994 |  |  |  |  |  |  |  |  |  |
| Kulikov 2016 | 0.7% | 2.2% |  |  |  |  |  |  |  |
| Kumar 1995 |  |  |  |  |  |  |  |  |  |
| Lee 2005 |  |  |  |  |  |  |  |  |  |
| Lee 2021 | 1.2% | 4.6% |  | 0.7% | 0.5% |  |  |  | 2.0% |
| Little 2009 |  |  |  |  |  |  |  |  |  |
| Lukac 2007 |  |  |  |  |  |  |  |  |  |
| Mar 2017 | 7.0% | 20.8% |  |  |  |  | 14.9% |  |  |
| Masaki 2018 | 2.6% | 6.6% | 0.8% | 0.8% | 2.8% | 3.1% |  |  | 1.9% |
| McCarthy 1993 |  |  |  | 0.3% |  |  |  |  |  |
| Millar 2000 |  |  |  |  |  |  |  |  |  |
| Nitta 1999 |  |  |  |  |  |  |  |  |  |
| Park 2018 | 0.3% | 0.4% |  | 1.4% |  |  | 0.1% |  |  |
| Pasic 2001 |  |  |  |  |  |  |  |  |  |
| Pasic 1998 |  |  |  |  |  |  |  |  |  |
| Patwardhan 2003 |  |  |  |  |  |  |  |  |  |
| Pozzoli 2014 |  |  |  | 0.5% | 0.0% |  |  | 0.0% |  |
| Robertson 2013 | 5.8% | 18.1% | 3.9% | 3.0% | 5.0% | 14.7% |  | 2.7% |  |
| Salmi 2020 | 20.4% | 71.2% |  | 17.6% |  | 55.6% | 28.3% | 7.4% |  |
| Sayed 2014 |  |  |  |  |  |  |  |  |  |
| Shin 2001 |  |  |  |  |  |  |  |  |  |
| Sie 2001 |  |  |  |  |  |  |  |  |  |
| Simms 2013 | 8.3% | 33.6% |  |  | 5.5% |  |  | 24.9% |  |
| Soga 2011 |  |  |  |  |  |  |  |  |  |
| Soni 2013 | 7.6% | 28.1% |  | 3.8% |  | 13.7% | 16.0% |  | 15.2% |
| Stulak 2011 |  |  |  |  |  |  |  |  |  |
| Sueda 2012 |  |  |  |  |  |  |  |  |  |
| Szalay 2004 |  |  |  |  |  |  | 3.2% |  |  |
| Szalay 1999 |  |  |  |  |  |  | 0.4% |  |  |
| Takeshita 1997 |  |  |  |  |  |  |  |  |  |
| Tamai 1995 |  |  |  |  |  |  |  |  |  |
| Tenpaku 2000 |  |  |  |  |  |  |  |  |  |
| Tuinenburg 2000 |  | 0.0% |  |  |  |  |  | 0.1% |  |
| Hemel 1997 |  |  |  |  |  |  |  |  |  |
| Hemel 1994 |  |  |  |  |  |  |  |  |  |
| Velimirovic 1997 |  |  |  |  |  |  |  |  |  |
| Viktorsson 2020 | 11.6% |  |  |  |  |  |  |  |  |
| Vogt 1997 |  |  |  |  |  |  |  |  |  |
| Waddingham 2021 | 53.6% |  |  |  |  |  |  |  |  |
| Wang 2013 |  |  |  | 0.4% |  |  |  |  |  |
| Yanagawa 2013 | 4.7% | 22.1% |  | 2.7% | 4.5% |  |  |  | 15.6% |
| Coti 2021 | 24.6% | 76.3% | 5.7% | 14.7% | 14.3% |  | 36.5% |  |  |
| MacGregor 2021 | 3.9% | 20.4% | 1.5% | 4.2% | 3.1% | 16.4% |  | 1.9% |  |
| Osmancik 2021 | 2.0% | 5.3% |  |  | 0.3% |  | 0.8% |  | 2.8% |
| Öner 2022 |  | 0.9% | 0.5% |  | 0.5% | 0.5% |  |  |  |
| Nitta 1999 |  |  |  |  |  |  |  |  |  |
| Ballaux 2006 |  |  |  | 0.7% |  |  |  |  |  |
| Patwardhan 2003 |  |  |  |  |  |  |  |  |  |
| Sayed 2014 |  |  |  |  |  |  |  |  |  |
| Tuinenburg 2000 |  | 0.7% |  |  |  |  |  | 0.7% |  |
| Velimirovic 1997 |  |  |  |  |  |  |  |  |  |
| Evtushenko 2015 |  |  |  |  |  |  |  |  |  |

Supplementary Table 3 - Quality of cohort and case-controls studies based on Newcastle Ottawa Scalce

| **study** | **study design** | 1) Representativeness of the exposed cohort | 2) Selection of the non-exposed cohort | 3) Ascertainment of exposure | 4) Demonstration that outcome of interest was not present at start of study | 1) Comparability of cohorts on the basis of the design or analysis | 1) Assessment of outcome | 2) Was follow-up long enough for outcomes to occur | 3) Adequacy of follow up of cohorts | total |
| --- | --- | --- | --- | --- | --- | --- | --- | --- | --- | --- |
| Pozzoli 2014 | cohort | 0 | 0 | 1 | 1 | 0 | 1 | 1 | 1 | 5 |
| Salmi 2020 | cohort | 1 | 0 | 1 | 1 | 2 | 1 | 1 | 1 | 8 |
| Szalay 2004 | cohort | 0 | 0 | 1 | 1 | 0 | 1 | 1 | 1 | 5 |
| Takeshita 1997 | cohort | 0 | 0 | 1 | 1 | 0 | 1 | 1 | 0 | 4 |
| Hemel 1997 | cohort | 0 | 0 | 1 | 1 | 0 | 1 | 1 | 1 | 5 |
| Hemel 1994 | cohort | 0 | 0 | 1 | 1 | 0 | 1 | 1 | 1 | 5 |
| Viktorsson 2020 | cohort | 1 | 0 | 1 | 1 | 0 | 1 | 1 | 1 | 6 |
| Waddingham 2021 | cohort | 1 | 0 | 1 | 1 | 2 | 1 | 1 | 1 | 8 |
| Wang 2013 | cohort | 1 | 0 | 1 | 1 | 0 | 1 | 1 | 1 | 6 |
| Mar 2017 | cohort | 1 | 0 | 1 | 1 | 0 | 1 | 1 | 1 | 6 |
| Masaki 2018 | cohort | 1 | 0 | 1 | 1 | 0 | 1 | 1 | 1 | 6 |
| Millar 2000 | cohort | 1 | 0 | 1 | 1 | 0 | 1 | 0 | 1 | 5 |
| Park 2018 | cohort | 1 | 0 | 1 | 1 | 0 | 1 | 1 | 1 | 6 |
| Pasic 2001 | cohort | 1 | 0 | 1 | 1 | 0 | 1 | 1 | 0 | 5 |
| Shin 2001 | cohort | 0 | 0 | 1 | 1 | 0 | 1 | 1 | 1 | 5 |
| Sie 2001 | cohort | 0 | 0 | 1 | 1 | 0 | 1 | 0 | 1 | 4 |
| Soga 2011 | cohort | 1 | 0 | 1 | 1 | 0 | 1 | 1 | 1 | 6 |
| Yanagawa 2013 | cohort | 1 | 0 | 1 | 1 | 0 | 1 | 1 | 1 | 6 |
| Jaeger 1994 | cohort | 1 | 0 | 1 | 1 | 0 | 1 | 1 | 1 | 6 |
| Izumoto 1998 | cohort | 1 | 0 | 1 | 1 | 1 | 1 | 1 | 1 | 7 |
| Al-Ghamdi 2016 | cohort | 1 | 1 | 1 | 0 | 1 | 1 | 1 | 0 | 6 |
| Bogachev-Prokophiev 2018 | cohort | 1 | 1 | 1 | 1 | 1 | 1 | 1 | 0 | 7 |
| Bogachev-Prokophieva 2012 | cohort | 1 | 1 | 1 | 1 | 1 | 1 | 1 | 0 | 7 |
| Chen 2005 | cohort | 1 | 1 | 1 | 1 | 1 | 1 | 1 | 0 | 7 |
| Cox 1993 | cohort | 1 | 1 | 1 | 1 | 1 | 1 | 1 | 0 | 7 |
| Feldman 1992 | cohort | 1 | 1 | 1 | 1 | 1 | 1 | 1 | 0 | 7 |
| Gelsomino 2016 | cohort | 1 | 1 | 1 | 1 | 1 | 1 | 1 | 1 | 8 |
| García-Villarreal 2003 | cohort | 1 | 1 | 1 | 1 | 1 | 1 | 1 | 0 | 7 |
| Arcidi 2000 | cohort | 1 | 1 | 1 | 1 | 1 | 1 | 0 | 0 | 6 |
| Ballaux 2006 | cohort | 1 | 1 | 1 | 1 | 1 | 1 | 1 | 1 | 8 |
| Rizzo 1996 | cohort | 1 | 1 | 1 | 1 | 1 | 1 | 1 | 0 | 7 |
| Herrmann 2019 | cohort | 1 | 1 | 1 | 1 | 1 | 1 | 1 | 1 | 8 |
| Izumoto 2000 | cohort | 1 | 1 | 1 | 1 | 0 | 1 | 1 | 1 | 7 |
| Jessurun 2000 | cohort | 1 | 1 | 1 | 1 | 0 | 1 | 1 | 0 | 6 |
| Kondo 2003 | cohort | 1 | 1 | 1 | 1 | 1 | 1 | 1 | 1 | 8 |
| Jouan 2016 | cohort | 1 | 1 | 1 | 1 | 0 | 1 | 1 | 1 | 7 |
| Coti 2021 | cohort | 0 | 0 | 0 | 0 | 0 | 0 | 0 | 0 | 0 |
| MacGregor 2021 | cohort | 0 | 0 | 0 | 0 | 0 | 0 | 0 | 0 | 0 |
| Osmancik 2021 | cohort | 1 | 0 | 1 | 1 | 1 | 1 | 1 | 1 | 7 |
| Bogachev-Prokophiev 2018 | cohort | 1 | 1 | 1 | 1 | 1 | 1 | 1 | 1 | 8 |
| Cho 2018 | cohort | 1 | 1 | 1 | 1 | 0 | 1 | 1 | 0 | 6 |
| Cox 1995 | cohort | 1 | 1 | 1 | 1 | 1 | 1 | 0 | 1 | 7 |
| Robertson 2013 | case-control | 1 | 1 | 0 | 1 | 0 | 1 | 1 | 1 | 6 |
| Ad 2017 | case-control | 1 | 1 | 0 | 1 | 1 | 1 | 1 | 1 | 7 |
| Evtushenko 2015 | case-control | 0 | 0 | 0 | 1 | 1 | 1 | 1 | 0 | 4 |
| Herry 2020 | case-control | 0 | 1 | 0 | 1 | 2 | 1 | 1 | 0 | 6 |
| Izzat 2020 | case-control | 0 | 1 | 0 | 1 | 1 | 1 | 1 | 0 | 5 |
| Itoh 2006 | case-control | 0 | 1 | 0 | 1 | 1 | 1 | 1 | 0 | 5 |
| Kim 2018 | case-control | 0 | 1 | 0 | 1 | 2 | 1 | 1 | 0 | 6 |
| Kho 2020 | case-control | 0 | 1 | 0 | 1 | 2 | 1 | 1 | 0 | 6 |
| Lukac 2007 | case-control | 0 | 1 | 0 | 1 | 1 | 1 | 1 | 0 | 5 |
| Jokinen 2009 | case-control | 0 | 1 | 0 | 1 | 2 | 1 | 1 | 0 | 6 |

Supplementary Table 4 - Quality of cross-sectional studies based on the modified Newcastle Otawwa Scale

| study | study design | Representativeness of the sample | Sample size | Non-respondents | Ascertainment of the exposure (risk factor) | The subjects in different outcome groups are comparable, based on the study design or analysis. Confounding factors are controlled | Assessment of the outcome | Statistical test: | total |
| --- | --- | --- | --- | --- | --- | --- | --- | --- | --- |
| Albåge 2004 | cross-sectional | 1 | 0 | 0 | 1 | 1 | 2 | 1 | 6 |
| Amirjamshidi 2020 | cross-sectional | 1 | 1 | 0 | 1 | 1 | 2 | 1 | 7 |
| Bis 2021 | cross-sectional | 1 | 1 | 1 | 1 | 1 | 2 | 1 | 8 |
| Cleveland 1972 | cross-sectional | 0 | 1 | 0 | 0 | 0 | 2 | 1 | 4 |
| Kon 1993 | cross-sectional | 1 | 0 | 1 | 1 | 0 | 2 | 1 | 6 |
| Guiraudon 1991 | cross-sectional | 1 | 0 | 0 | 1 | 0 | 2 | 1 | 5 |
| Kulikov 2016 | cross-sectional | 1 | 0 | 0 | 1 | 1 | 2 | 1 | 6 |
| Little 2009 | cross-sectional | 1 | 0 | 0 | 1 | 0 | 2 | 1 | 5 |
| Kim 1999 | cross-sectional | 1 | 0 | 0 | 1 | 0 | 2 | 1 | 5 |

Supplementary Table 5 - Quality of non-randomised clinical trials based on ROBINS-I tool

| **Study** | **Study design** | **Bias due to confounding** | **Bias in selection of participants into the study** | **Bias in classification of interventions** | **Bias due to deviations from intended interventions** | **Bias due to missing data** | **Bias in measurement of outcomes** | **Bias in selection of the reported result** | **Overall bias** |
| --- | --- | --- | --- | --- | --- | --- | --- | --- | --- |
| Soni 2013 | nRCT | Moderate | low | Moderate | Serious | NI | low | Serious | Moderate |
| Stulak 2011 | nRCT | Serious | low | low | low | low | low | NI | low |
| Szalay 1999 | nRCT | Serious | low | Serious | low | Serious | low | NI | Serious |
| Tenpaku 2000 | nRCT | serious | low | serious | moderate | low | low | low | moderate |
| Tuinenburg 2000 | nRCT | moderate | low | moderate | low | low | low | low | low |
| Velimirovic 1997 | nRCT | moderate | low | moderate | low | moderate | low | moderate | moderate |
| Nitta 1999 | nRCT | moderate | low | low | low | low | low | low | low |
| Patwardhan 2003 | nRCT | serious | low | moderate | low | low | low | low | moderate |
| Simms 2013 | nRCT | serious | low | moderate | low | low | low | low | moderate |
| Lee 2021 | nRCT | low | moderate | NI | low | low | low | low | low |

Supplementary Table 6 - Quality of randomised clinical trials based on RoB2

| **study** | **study design** | **Bias arising from the randomisation process** | **Bias due to deviations from intended interventions** | **Bias due to missing outcome data** | **Bias in measurement of the outcome** | **Bias in selection of the reported result** | **Overall bias** |
| --- | --- | --- | --- | --- | --- | --- | --- |
| Sayed 2014 | RCT | low | some concerns | low | some concerns | low | some concerns |

Supplementary Table S 7 - Quality of included case-series based on JBI instrument

| study | study design | Were there clear criteria for inclusion in the case series? | Was the condition measured in a standard, reliable way for all participants included in the case series? | Were valid methods used for identification of the condition for all participants included in the case series? | Did the case series have consecutive inclusion of participants? | Did the case series have complete inclusion of participants? | Was there clear reporting of the demographics of the participants in the study? | Was there clear reporting of clinical information of the participants? | Were the outcomes or follow up results of cases clearly reported? | Was there clear reporting of the presenting site(s)/clinic(s) demographic information? | Was statistical analysis appropriate? | total |
| --- | --- | --- | --- | --- | --- | --- | --- | --- | --- | --- | --- | --- |
| Sueda 2012 | case-series | 1 | 1 | 1 | 0 | 0 | 1 | 0 | 1 | 0 | 1 | 6 |
| McCarthy 1993 | case-series | 0 | 1 | 1 | unclear | unclear | 1 | 1 | 1 | 0 | 1 | 6 |
| Hurlé 2004 | case-series | 0 | unclear | 1 | 0 | unclear | 1 | 1 | 1 | 0 | 0 | 4 |
| Hemels 2006 | case-series | 1 | 1 | 1 | 1 | 1 | 1 | 1 | 1 | 0 | 1 | 9 |
| Kawashima 1998 | case-series | 1 | 1 | 1 | 1 | 1 | 0 | 1 | 1 | 0 | 1 | 8 |
| Koda 2019 | case-series | 1 | 1 | 1 | 1 | 1 | 0 | 1 | 1 | 0 | 1 | 8 |
| Khargi 2004 | case-series | 1 | 1 | 1 | 1 | 1 | 0 | 1 | 1 | 0 | 1 | 8 |
| Lee 2005 | case-series | 1 | 1 | 1 | 1 | 1 | 0 | 1 | 1 | 0 | 1 | 8 |
| Albåge 2000 | case-series | 1 | 1 | 1 | 1 | 1 | 1 | 1 | 1 | 1 | 0 | 9 |
| Kosakai 1994 | case-series | 1 | 1 | 1 | 1 | 1 | 1 | 1 | 1 | 1 | 1 | 10 |
| Öner 2022 | case-series | 1 | 1 | 0 | 1 | 1 | 1 | 1 | 1 | 1 | 1 | 9 |

Supplementary Table 8 - characteristics of studies reporting sinus node dysfunction

| **Study** | **Study Design** | **Interventions** | **follow-up** | **N** | **mean Age (SD)** | **Gender (female)** | **mean CPB time (SD)** | **mean XCL time (SD)** | **SND number** | **SND prevalence** |
| --- | --- | --- | --- | --- | --- | --- | --- | --- | --- | --- |
| Kim 2018 | case-control | valve, other | 27 ± 24 months | 202 | 56 (11.5) | 61.39% | 147.08 (63.4) | 105.9 (32.5) | 37 | 18.32% |
| Sayed 2014 | RCT | valve, other | mean 56 ± 12 months | 40 | 35.3 (9.12) | 52.50% | - | - | 2 | 5.00% |
| Velimirovic 1997 | nRCT | valve, other | 13.9 ± 8 months | 12 | 57.3 (7.5) | 83.33% | 120 (7.8) | 95 (4.4) | 0 | 0.00% |
| Velimirovic 1997 | nRCT | valve, other | 13.9 ± 8 months | 10 | 51.5 (6.7) | 70.00% | 75 (6.3) | 45 (3.4) | 0 | 0.00% |
| Pasic 1998 | case-series | valve, maze, other | 1 year | 15 | 62 (0) | 86.67% | - | - | 12 | 80.00% |
| Tamai 1995 | case-series | valve, maze, other | 1 year | 25 | 57 (8) | 40.00% | 210 (47) | 137 (32) | 1 | 4.00% |
| Chen 2005 | cohort | valve, maze, CABG, other | 46.5 ± 24 months | 71 | 50.8 (11) | 53.52% | - | - | 2 | 2.82% |
| Szalay 1999 | nRCT | valve, maze, CABG | 3.6 ± 0.9 months (range 2.5 - 7 months) and 14.9 ± 2.2 months (range 12-19 months) postoperatively | 52 | 64.4 (9.5) | 59.62% | 143.46 (51.38) | 92.38 (27.26) | 38 | 73.08% |
| Soga 2011 | cohort | valve, CABG, other | up to 72 months (mean 30 months) | 66 | 64.88 (2.27) | 37.88% | 116.21 (7.47) | 90.29 (6.97) | 18 | 27.27% |
| Bogachev-Prokophiev 2018 | cohort | Valve, CABG, Maze, other | 37.3 ± 34.1 months | 312 | 54.85 (9.93) | 38.46% | 114.5 (77.31) | 84.75 (54.4) | 17 | 5.45% |
| Al-Ghamdi 2016 | cohort | valve, CABG | mean of 84.5 ± 30 months ranging from 35 to 109 months | 1234 | 46.65 (16) | 41.00% | 126.02 (66.85) | 89.58 (48.48) | 3 | 0.24% |
| Herrmann 2019 | cohort | valve, CABG | maximum follow-up of 14.2 years and a median follow up time of 4.0 years | 505 | 72 (0) | 57.62% | 145 (0) | 91 (0) | 97 | 19.21% |
| Izzat 2020 | case-control | valve, CABG | - | 319 | 44.17 (17.08) | 55.80% | 115.59 (39.34) | 86.97 (30.09) | 8 | 2.51% |
| Patwardhan 2003 | nRCT | valve, CABG | 6 months | 64 | 33 (12.9) | 64.06% | 88.7 (36.3) | 54.9 (21.7) | 0 | 0.00% |
| Albåge 2004 | cross-sectional | maze, valve, other | mean follow-up time of 45 (range 16–93) months | 37 | 54 (10) | 35.14% | - | - | 6 | 16.22% |
| Albåge 2000 | case-series | maze, valve, CABG, other | 3–55 months (median 18 months) | 26 | 55 (0) | 26.92% | 175 (0) | 93 (0) | 5 | 19.23% |
| Arcidi 2000 | cohort | maze, valve, CABG, other | 3 months | 99 | 61.08 (10.22) | 45.45% | - | - | 10 | 10.10% |
| Izumoto 2000 | cohort | maze, valve, CABG, other | 44.6 ± 1.1 months | 100 | 59.7 (0) | 55.00% | - | - | 10 | 10.00% |
| Izumoto 1998 | cohort | maze, valve, CABG, other | - | 87 | 59.3 (10.2) | 59.77% | 177.2 (70.1) | 121.7 (30.8) | 7 | 8.05% |
| Cho 2018 | cohort | maze, valve, CABG | median follow-up 3.6 (interquartile range, 1.7–6.4) years | 750 | 56.8 (12.2) | 44.53% | 167.2 (55.2) | 108.7 (45.5) | 35 | 4.67% |
| Kim 1999 | cross-sectional | maze, valve, CABG | 30 ± 13 months (12–56 months) | 75 | 48 (10) | 66.67% | 251 (73) | 151 (43) | 4 | 5.33% |
| Lee 2005 | case-series | maze, valve, CABG | - | 13 | 64 (8.32) | 46.15% | 156.67 (28.35) | 101.83 (18.91) | 1 | 7.69% |
| Millar 2000 | cohort | maze, valve, CABG | 5 years (1993 to 1998) | 76 | 59.6 (10) | 40.79% | 188.23 (49.1) | 107.19 (40.19) | 9 | 11.84% |
| Pasic 2001 | cohort | maze, valve, CABG | follow-up period ranged from 1 to 11 months (mean, 4 months) | 48 | 64 (10) | 58.33% | 110 (32) | 64 (17) | 36 | 75.00% |
| Cox 1995 | cohort | maze, unknown | 3 months | 123 | 52.7 (11) | 26.02% | - | - | 12 | 9.76% |
| Ballaux 2006 | cohort | maze, unknown | mean 4.0 ± 2.6 years | 64 | 58.1 (10.9) | 34.38% | 148 (30.6) | 90 (22.5) | 3 | 4.69% |
| Bogachev-Prokophiev 2018 | cohort | maze, other | 23.7 ± 1.3 months | 45 | 52.8 (14.2) | 62.22% | - | 61 (36) | 0 | 0.00% |
| Evtushenko 2015 | case-control | maze, CABG | 42.2 ± 10.4 months | 141 | 52.9 (9.5) | 53.90% | - | - | 58 | 41.13% |
| Ballaux 2006 | cohort | maze | 4.0 ± 2.6 years | 139 | 50.7 (8.7) | 14.39% | 114 (18.5) | 61 (18.1) | 6 | 4.32% |
| Jessurun 2000 | cohort | maze | 31 ± 16 months | 41 | 49 (8) | 14.63% | - | - | 6 | 14.63% |
| Hemel 1997 | cohort | corridor procedure | 48 ± 35 months | 30 | 53 (10) | 33.33% | - | - | 11 | 36.67% |
| Hemel 1994 | cohort | corridor procedure | 41 ± 16 months | 36 | 55 (11) | 30.56% | 136 (24) | 82 (8) | 10 | 27.78% |
| Amirjamshidi 2020 | cross-sectional | valve | - | 359 | 68 (11) | 37.33% | 125 (42) | 93 (25) | 62 | 17.27% |
| Guiraudon 1991 | cross-sectional | valve | - | 34 | - | 52.94% | - | 45 (0) | 0 | 0.00% |
| Herry 2020 | case-control | valve | - | 924 | 73.23 (9.41) | 31.60% | 80.75 (0) | 63.11 (0) | 8 | 0.87% |
| Kumar 1995 | nRCT | valve | 6 weeks | 89 | 34.88 (0) | 53.93% | 91.79 (0) | 67.27 (0) | 28 | 31.46% |
| Shin 2001 | cohort | valve | mean 34 ± 24 months (ranged from 3 to 87 months) | 46 | 62 (121) | 60.87% | - | - | 6 | 13.04% |
| Takeshita 1997 | cohort | valve | 12-26 months | 76 | 56.08 (10.97) | 0.00% | - | - | 16 | 21.05% |
| Tuinenburg 2000 | nRCT | valve | Median follow-up dura- tion was 17 months (range 3 to 26) | 32 | 56.78 (11.85) | 65.63% | 128.46 (40.78) | 72.09 (21.33) | 0 | 0.00% |
| Patwardhan 2003 | nRCT | valve, maze | 6 months | 84 | 32.5 (10.95) | 48.81% | 96.1 (24) | 52.8 (15.3) | 1 | 1.19% |
| Tuinenburg 2000 | nRCT | valve, maze | Median follow-up dura- tion was 17 months (range 3 to 26) | 13 | 57 (12) | 53.85% | 145 (28) | 90 (23) | 0 | 0.00% |
| Vogt 1997 | case-series | valve, maze | 7.5 + 5.2 months | 17 | 63 (12) | 0.00% | - | - | 2 | 11.76% |
| Sayed 2014 | RCT | valve, maze | mean 56 ± 12 months | 40 | 22.4 (11.5) | 57.50% | - | - | 4 | 10.00% |
| Kawashima 1998 | case-series | maze, valve | 2.12 ± 0.85 years | 30 | 57.4 (11) | 46.67% | 244 (37) | 158 (33) | 3 | 10.00% |
| Kosakai 1994 | case-series | maze, valve | follow-up times ranging from 6 to 25 months, | 62 | 56.3 (0) | 51.61% | 226 (34) | 142 (25) | 2 | 3.23% |
| Kulikov 2016 | cross-sectional | maze, valve | - | 100 | 59.3 (10.2) | 52.00% | - | - | 12 | 12.00% |
| McCarthy 1993 | case-series | maze, valve | 1 year | 14 | 48 (10) | 35.71% | 143 (18.5) | 69 (14.5) | 14 | 100.00% |
|  |  |  |  |  | **56.95 (11.19)** | **44.48% (11.70)** | **129.39 (35.12)** | **88.15 (20.08)** | **13.71% (95% CI 8.13 - 20.33)** | |
